# Self-supporting carbon-rich SiOC ceramic electrodes for lithium-ion batteries and aqueous supercapacitors†‡

**DOI:** 10.1039/d1ra05968h

**Published:** 2021-11-03

**Authors:** Shakir Bin Mujib, François Ribot, Christel Gervais, Gurpreet Singh

**Affiliations:** Department of Mechanical and Nuclear Engineering, Kansas State University Manhattan KS 66506 USA gurpreet@ksu.edu; Sorbonne Université, CNRS UMR 7574, Laboratoire de Chimie de la Matière Condensée de Paris 75005 Paris France

## Abstract

Fabrication of precursor-derived ceramic fibers as electrodes for energy storage applications remains largely unexplored. Within this work, three little known polymer-derived ceramic (PDC)-based fibers are being studied systemically as potential high-capacity electrode materials for electrochemical energy devices. We report fabrication of precursor-derived SiOC fibermats *via* one-step spinning from various compositions of siloxane oligomers followed by stabilization and pyrolysis at 800 °C. Electron microscopy, Raman, FTIR, XPS, and NMR spectroscopies reveal transformation from polymer to ceramic stages of the various SiOC ceramic fibers. The ceramic samples are a few microns in diameter with a free carbon phase embedded in the amorphous Si–O–C structure. The free carbon phase improves the electronic conductivity and provides major sites for ion storage, whereas the Si–O–C structure contributes to high efficiency. The self-standing electrodes in lithium-ion battery half-cells deliver a charge capacity of 866 mA h g_electrode_^−1^ with a high initial coulombic efficiency of 72%. As supercapacitor electrode, SiOC fibers maintain 100% capacitance over 5000 cycles at a current density of 3 A g^−1^.

## Abstract 2

Fabrication of precursor-derived ceramic fibers as electrodes for energy storage applications remains largely unexplored.

## Graphical abstract

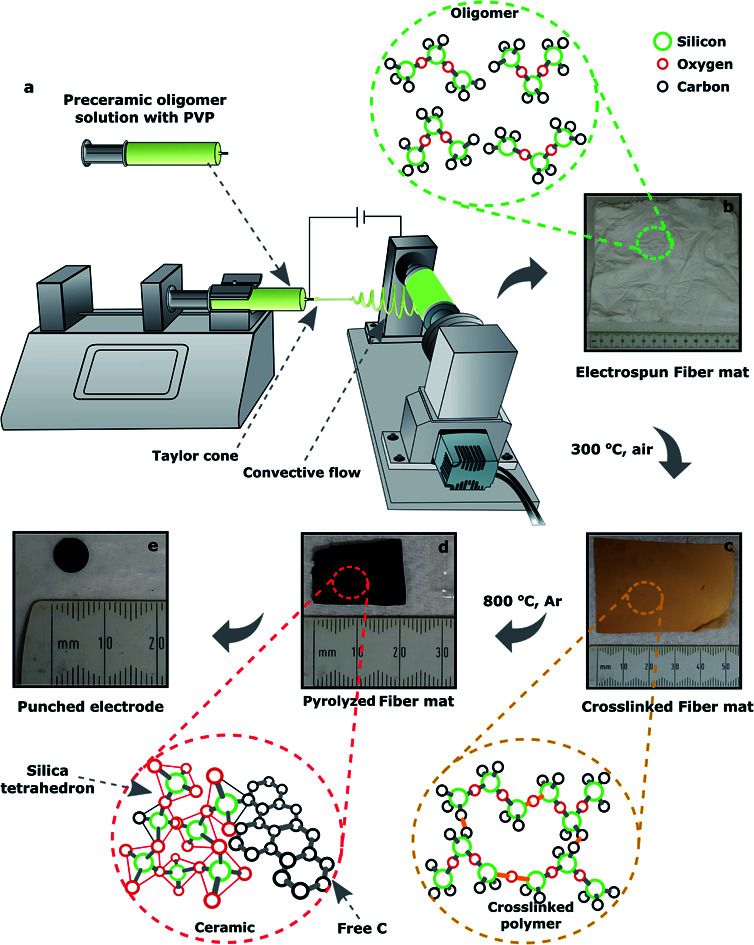

## 1. Introduction

Despite the exponential rise in research activity in the design and development of micro-/nano-structured electrode materials for electrochemical energy storage devices,^1–4^ graphite or carbon-coated metal foil remain the electrode of choice for most capacitors and Li-ion battery (LIB) technologies. Materials with higher charge storage than traditional carbons such as silicon [almost 10× lithium (Li) storage capacity (∼4.2 A h g^−1^)] emerged as potential replacements for graphite owing to silicon's large abundance in the earth's crust, low toxicity, and well-established manufacturing technology for its large scale production.^5–7^ However, drastic volume variation and pulverization during charge/discharge, excessive formation of solid electrolyte interfaces (SEIs), and stress-induced cracks in Si electrodes has rendered bulk silicon unusable as an electrode for LIBs.^6,8^

Efforts related to the development and use of nanostructured silicon-based electrodes (utilizing the size effect to achieve fast Li-ion transport, and prevent fracture propagation in electrodes), in the form of nanoparticles,;^9–12^ nanotubes and nanowires,^13–17^ and composite nanoparticles with carbon^18–23^ are now well-documented. Nevertheless, capacity decay over a long period of charge/discharge, high first cycle loss, high cost of material synthesis, and challenges associated with scalability limit their applications as electrodes for commercial applications.

To circumvent the issues associated with silicon electrodes, silicon oxycarbide (SiOC) derived from pyrolysis of ceramic precursor polymers has renewed attention in recent years from electrochemical storage point of view.^24–33^ Potential of polymer-derived ceramic (PDC) (*e.g.*; SiOC, SiCN) materials in energy storage, initially proposed by Dahn *et al.* in 1990s, largely depends on the chemical structures of the material.^34,35^ SiOC is an amorphous ceramic mainly consists of a Si–O–C glass phase with a free carbon region, where the mixed bonds of Si with O and C enable high Li storage.^36,37^ According to Graczyk-Zajac *et al.*, mixed bonds in SiOC allows more disordered carbon phase for higher Li capacity (325 mA h g^−1^) as well as reversible storage sites at the interface of the carbon phase and amorphous network.^38^ Kasper *et al.* showed that the electrical conductivity of SiOC samples improved (0.07 to 2.2 S m^−1^) with the increasing amount of free carbon phase, in addition to enabling more active sites for Li.^39^ The edges of the free carbon phase and the interstitial spaces are major electrochemically active sites for Li ions, whereas micro-/nano-voids and amorphous Si–O–C network contribute to minor Li storage.^25,40,41^ Lee *et al.* reported that controlling the nanovoids in the Si–O–C domain derived from PSS-octakis(dimethylsilyloxy)silsesquioxane (POSS) provided efficient ion pathway and withstood structural degradation by buffering during lithiation/delithiation.^33^ As a result, the authors were able to achieve a capacity of 412 mA h g^−1^ at a high current density of 3600 mA g^−1^. Furthermore, pyrolysis parameters are always crucial in controlling the microstructure (*e.g.*; free/disordered carbon phase and nanovoids) and thus maintaining the electrochemical properties of SiOC. For example, pyrolysis temperature above 900 °C results in lower capacity and unstable cycling behavior of the SiOC ceramics compared to lower temperature.^29,42^ With increasing temperature disordered carbon phase arranges into ordered structure and amorphous Si–O–C phase decomposes to electrochemically inactive SiC crystals. In addition, metal foil-based electrode, conventionally prepared using a doctor blade, carries the inactive weight of polymeric binders, conducting agents (usually carbon black), and the foil itself, which do not contribute towards electrode capacity.^31^

Since the development of electrospinning in 1930s by Formhlas,^43,44^ the technique has been explored to fabricate a variety of artificial fibers from homogenous polymer solution using high potential electric field. Nanofibers produced by electrospinning can provide large surface area, exceptional tolerance against mechanical deformation with improved electrical conductivity and enhanced electrochemical performance. For example, electrospinning of PAN nanofibers with Si nanoparticles delivered a flexible electrode after carbonization that showed a high capacity of 1600 mA h g^−1^ in LIBs.^45^ Xiao *et al.* reported a stretchable PVDF nanofiber membrane which was coated with Ni and Si to form a Si/Ni/PVDF core–shell structure.^46^ This soft silicon electrode showed 56.9% capacity retention after 1000 cycles with 20% stretching capability.

Herein, we report on the fabrication of freestanding, binder-free SiOC electrodes by an electrospinning method using liquid precursor polymer, followed by thermal treatment and pyrolysis. Three new types of SiOC precursors, that are readily available, and easy to draw into low-cost, high-yield ceramic fibers compared to commercial ceramic fibers,^47,48^ are utilized in this study both for LIBs and supercapacitors. To the best of our knowledge such siloxanes have not been reported before for the fabrication of SiOC ceramic fibermats, that are investigated in electrochemical devices. Scanning- and transmission-electron microscopy reveals uniform, defect-free amorphous structure of the SiOC fibers. Fourier transform infrared spectroscopy (FTIR) and nuclear magnetic resonance (NMR) are utilized to study the polymeric precursor to ceramic transformation of the siloxanes, whereas Raman spectroscopy and X-ray photoelectron spectroscopy (XPS) confirms the presence of “free carbon” content. Amorphous Si–O–C phase provides chemical stability and host sites for Li storage, and free carbon phase contributes to conductive network as well as the high Li capacity of the fibermat. As a result, as-prepared SiOC electrodes deliver specific capacity as high as 866 mA h g_electrode_^−1^ (considering total weight of the electrode) with high first cycle coulombic efficiency of 72% in LIBs. Specific capacity of 800 mA h g^−1^ at a current density of 50 mA g^−1^ is achieved with a 100% capacity retention after 50 cycles for a particular SiOC electrode. Further, as supercapacitor electrode SiOC fibers have delivered a high gravimetric capacitance of 55 F g^−1^ at 100 mV s^−1^ and maintained 100% capacitance over 5000 cycles at 3 A g^−1^.

## 2. Experimental

### 2.1 Materials

**Fig. 1:**
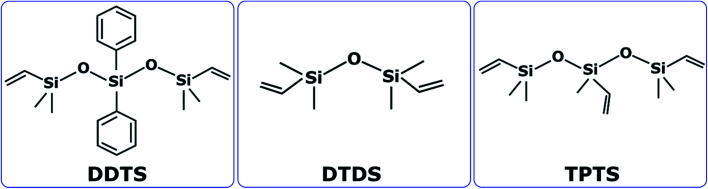
Molecular structure of three pre-ceramic silicon oligomers used in this study.

Three types of commercially available oligomers precursors to silicon oxycarbide ceramic were sourced from Gelest, Inc. (Pennsylvania, USA). These SiOC precursors: (1) 1,5-divinyl-3,3-diphenyl-1,1,5,5-tetramethyltrisiloxane (cited as DDTS), (2) 1,3-divinyltetramethyldisiloxane (cited as DTDS), and (3) 1,3,5-trivinyl-1,1,3,5,5-pentamethyltrisiloxane (cited as TPTS) as shown in Fig. 1 have comparable chemical structures with vinyl groups which are potential crosslinking groups. Fig. 1 shows the compositions of the precursors where silicon (Si) to oxygen (O) atom ratio for DDTS and TPTS is 3 : 2 and for DTDS is 2 : 1.

To accomplish electrospinning of the preceramic oligomers 10 wt% of polyvinylpyrrolidone (PVP) with *M*_w_ ≈ 1 300 000 g mol^−1^ (Sigma Aldrich, Missouri, USA) was used as a spinning agent. Dicumyl peroxide (Sigma Aldrich, Missouri, USA) was used as a crosslinker for the oligomers and iso-propanol (Fisher Scientific, Massachusetts, USA) was used as a solvent. Ultra-high purity argon (Ar) gas was used for inert environment during pyrolysis which was supplied by Matheson (Kansas, USA).

### 2.2 Electrospinning and synthesis of fibermat

**Fig. 2:**
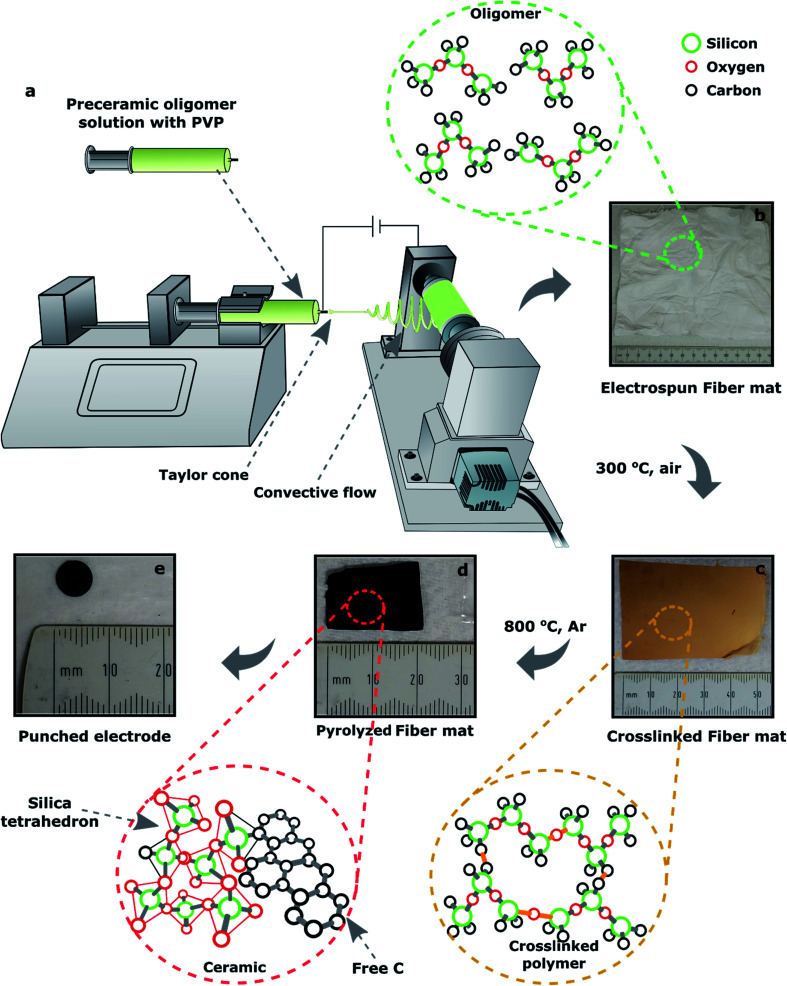
Fabrication of SiOC ceramic fibermats *via* electrospinning process. (a) Electrospinning set-up, (b) as-spun fibermat collected to from the roller, (c) fibermat after crosslinking at ∼300 °C in air, (d) fiber mat after pyrolysis at ∼800 °C in Ar environment, and (e) a 6.35 mm dia. electrode punched out from the ceramic fibermat.

Various SiOC fibermat samples were fabricated *via* electrospinning of polymer solutions, followed by stabilization and pyrolysis at elevated temperatures. The electrospinning setup that was designed and built in the lab consisted of four major parts:^47^ (1) a syringe which is controlled with a stepper motor works as a feeder for polymer solution; (2) a voltage source (up to 30 kV) that controls the amount of electrical charge applied to the solution; (3) an aluminum roller that works as a fibermat collector; and (4) a microcontroller made of Arduino® that controls the motors' speed and the sensors. The major parts of the setup are illustrated in Fig. 2.

The electrospinning solution was prepared by dissolving 10 wt% of PVP in 7500 mg of iso-propanol; after that preceramic oligomer was added into the solution with a weight ratio of 3 : 1 to PVP. DCP as a crosslinking catalyst was already dissolved into the preceramic oligomer, which was 1 wt% of the siloxane oligomer. The solution was then stirred for about 2 h to get a homogenous mixture of PVP and preceramic oligomer. The solution was then poured into the feeder syringe. During electrospinning, the feed rate was set at 5 mL h^−1^ while the voltage supply was maintained at 15 kV. The positive output was connected to the syringe nozzle and the collector was grounded. The nozzle to collector distance was set to 25 cm. The collector was wrapped with an Al foil and a fibermat of 15 × 15 cm^2^ was collected onto the foil after the electrospinning. The electrospun raw or as-spun fibermat is shown in Fig. 2b.

### 2.3 Crosslinking and pyrolysis of the fibermat

The electrospun fibermats of the three siloxanes were dried at room temperature overnight and then crosslinked at ∼300 °C in an oven for 8 h in the presence of air. The crosslinked fibermats (Fig. 2c) were then pyrolyzed in an alumina tube furnace at approx. 800 °C for 30 min in flowing Ar gas. The heating rate was approx. 2 °C min^−1^ and Ar gas flow rate was 5 mL min^−1^ in the furnace. The crosslinked fibermats were cut into smaller rectangular shape (50 × 25 mm^2^) to fit in the aluminum ceramic boat that was used to hold the samples in the furnace during pyrolysis. The fibermat polymer-to-ceramic conversion was complete at 800 °C; a representative of SiOC ceramic fibermat is shown in Fig. 2d. The lateral shrinkage during polymer-to-ceramic conversion for DDTS-derived SiOC ceramic fibermat was 40%, whereas for DTDS- and TPTS-derived SiOC ceramic fibermats were 60% and 55% respectively.

### 2.4 Characterization techniques

Several material characterization techniques were employed to determine the morphological, compositional, and chemical conversions at various stages of processing of the fibers-as-spun, crosslinked, and pyrolyzed fibermats. The images of surface features, shapes, and diameters of the fibers in the SiOC ceramic fibermats were investigated using a FEI Nova NanoSEM 450 scanning electron microscope (SEM). An FEI Tecnai Osiris 200 kV (scanning) transmission electron microscope (TEM) was used to obtain morphological and crystallographic information as well as energy dispersive X-ray (EDX) mapping analysis of the ceramic fibermats.

To determine the molecular structure and chemical interactions of the ceramic fibermats Raman spectroscopy and Fourier-transform infrared spectroscopy (FTIR) were used. Raman analysis in the range of 800–2000 cm^−1^ was performed to determine mainly the carbon vibrational modes using a confocal micro-Raman microscope (Horiba Jobin Yvon LabRam ARAMIS) equipped with a HeNe laser source (632.8 nm). The presence and evolution of various chemical functional groups of the siloxane precursors in the fibermats were investigated using a PerkinElmer Spectrum 400 FTIR spectrometer in the range of 500–3500 cm^−1^.

To further investigate the chemical bonds in each sample, nuclear magnetic resonance (NMR) spectroscopy was used. A Bruker AVANCE 300 spectrometer was used to record solid-state ^13^C CP MAS and ^29^Si MAS NMR spectra using following specifications (*B*_0_ = 7.0 T, *υ*_0_(^1^H) = 300.29 MHz, *υ*_0_(^13^C) = 75.51 MHz, *υ*_0_(^29^Si) = 59.66 MHz), and a 4 mm Bruker probe and spinning frequency of 10 kHz. ^13^C CP MAS experiments were recorded with ramped amplitude cross-polarization in the ^1^H channel to transfer magnetization from ^1^H to ^13^C (recycle delay = 3 s, CP contact time = 1 ms, optimized ^1^H spinal-64 decoupling). Single-pulse ^29^Si NMR MAS spectra were recorded with recycle delays of 60 s. Liquid-state NMR spectra were recorded on a Bruker AVANCE 300 spectrometer (*B*_0_ = 7.0 T, *υ*_0_(^1^H) = 300.13 MHz, *υ*_0_(^13^C) = 75.47 MHz, *υ*_0_(^29^Si) = 59.63 MHz). Chemical shift values were referenced to tetramethyl silane for ^13^C and ^29^Si.

X-ray photoelectron spectroscopy (XPS) was carried out to analyze the surface composition using a Thermo Scientific Al Kα^+^ ion beam (beam energy = 1486.6 eV and spot size = 400 μm) XPS. To remove the surface contamination of the ceramic fiber mats, initial sputtering of the surface with Ar^+^ at 3.0 keV for 2 min was performed.

### 2.5 Electrode preparation and electrochemical measurement

Ceramic fibermats were tested electrochemically both as organic electrolyte-based lithium-ion batteries (LIBs) and aqueous supercapacitors electrodes. Electrospun SiOC ceramic fibermats were used as freestanding electrodes in LIBs half-cells. A disk electrode was punched out from the pyrolyzed fibermat (Fig. 2e) with diameter of about 6.35 mm (1/4 inch), which was used as the working electrode. A glass fiber membrane (*φ* ≈ 19 mm, *t* ≈ 25 μm) (GE, USA) as separator, pure Li metal (*φ* ≈ 14.3 mm, *t* ≈ 75 μm) (Alfa Aesar, USA) as the counter electrode, and approximately 6 drops of 1 M lithium hexafluorophosphate (LiPF_6_) in (1 : 1 v/v) dimethyl carbonate (DMC) : ethylene carbonate (EC) (Sigma Aldrich, USA) as the electrolyte were used. The cells were assembled in LIR 2032 coin cells in a glove box, and the assembled cells were tested using a multichannel BT2000 Arbin test system (Texas, USA) between 10 mV to 2.5 V *vs.* Li/Li^+^. The cells were subjected to symmetric cycling at current densities of 50, 100, 200, 400, 800, 400, 200, 100, 50 mA g^−1^ for 5 cycles each.

For supercapacitor testing, a three-electrode setup was used. Ceramic fibermats (as an active material) were mixed with 10 wt% carbon black (as a conducting agent) (Alfa Aesar, Massachusetts, USA) and 5 wt% polyvinylidene fluoride (PVDF) (as a binder) (Sigma Aldrich, Missouri, USA) thoroughly; approximately 4–6 drops of *N*-methyl 2 pyrrolidone (NMP) (Alfa Aesar, Massachusetts, USA) was also added to form a slurry of uniform consistency. The slurry was then pasted on stainless steel (SS) mesh (1 × 1 cm^2^) using a flat paint brush, followed by drying at 80 °C overnight in an oven. The ceramic coated SS was then used as working electrode in the three-electrode setup, where Pt wire and Ag/AgCl were used as the counter and reference electrode, respectively. 1 M NaCl was used as an electrolyte. A CHI 660 electrochemical workstation (CH Instruments, Inc., Texas, USA) was used to perform cyclic voltammetry (CV) and galvanostatic charge–discharge (GCD) of the electrodes. CV and GCD were performed in the potential window of 0–1 V at various scan rates and current densities, respectively. Electrochemical impedance spectroscopy (EIS) was also carried out from 0.01 Hz to 100 kHz at an amplitude of 5 mV. For CV and GCD, the specific capacitance values were calculated using eqn (1) and (2), respectively.^49^1
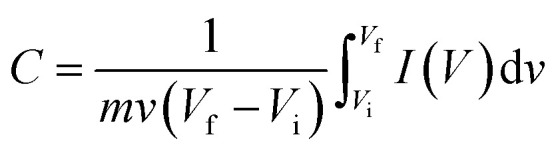
2
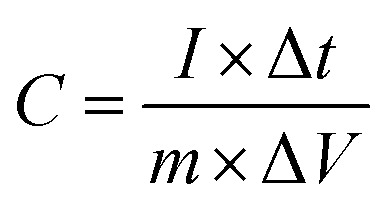
where, *m* is the active mass of electrode, *v* is the scan rate, *V*_i_ and *V*_f_ are the initial and final voltage, *I*(*V*) is response current density, *I* is charge/discharge current, and Δ*V* is the potential window.

## 3. Results and discussions

### 3.1 Structure and morphology of fibermats

**Fig. 3:**
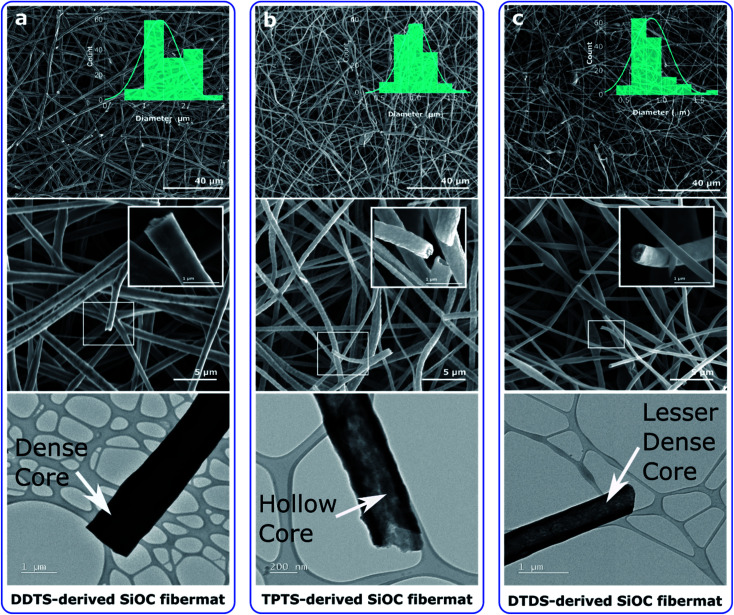
SEM (top and middle) and corresponding TEM (bottom) images of the electrospun SiOC fibermats. (a–c) The images reveal the formation of thick, smooth, and dense fibers for the SiOC samples produced from preceramic Si oligomers with high molecular weight compared to low molecular weights.

SEM and TEM images of the pyrolyzed samples revealed the surface and internal features of the SiOC fibers. Fig. 3 shows that SiOC fibermats fabricated from the three siloxane oligomer samples were largely uniform in diameter along the fiber length. For DDTS-derived SiOC fibers the average fiber diameter was between 1–3 μm, whereas for DTDS- and TPTS-derived SiOC fibers the average dimeter stayed between 0.2–1.5 μm. SiOC fibers derived from the siloxane oligomer with high molecular weight resulted in large diameter fibers compared to low molecular weight precursors. Furthermore, high molecular weight precursor produced thick and dense fibers as can be seen from the inset of the high-resolution SEM images (Fig. 3a). The thinner and hollow fibers derived from the DTDS and TPTS may have resulted from the steric hindrance, and molecular weight difference with the PVP (spinning agent) during the spinning process.^47^ As a result, in our case of single-nozzle electrospinning, the PVP molecules moved to the inner layers and preceramic polymer stayed in the outer layers, and the separation likely occurred during the crosslinking and pyrolysis processes.^50^ The core–shell structure of the hollow TPTS-derived SiOC fibers was also confirmed by TEM. In addition, SEM and TEM revealed a smoother surface for DDTS- and DTDS-SiOC compared to TPTS-SiOC. The hollow fibers with bead-on-string structure of the TPTS-SiOC was attributed to the low molecular weight as well as the concentration and viscosity of the spinning solution.^51–53^ High-resolution TEM images of the pyrolyzed fibermats, illustrated in Fig. S1,‡ suggested the amorphous or featureless structure of the three fiber types. The results of the EDX elemental analyses, also shown in Fig. S1,‡ confirmed the homogenous distribution of the Si, O, and C elements in the ceramic fibers.

### 3.2 Compositional analysis

**Fig. 4:**
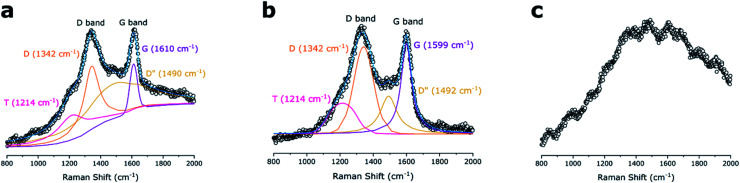
Comparison of Raman spectra for the SiOC samples. (a) DDTS- and (b) DTDS-derived SiOC samples show integrated carbon D and G bonds, whereas (c) TPTS-derived SiOC show strong fluorescence.

Raman spectra (Fig. 4) show the presence of carbon domains which allows to evaluate corresponding microstructures in the ceramic materials. The D and G vibrational bands at 1342 and ∼1600 cm^−1^ for the DDTS- and DTDS-derived SiOC, suggested the presence of “free carbon” structures *i.e.*, carbon bonded to carbon and not any other elements in the ceramic. The intense D bands suggested the existence of disordered aromatic rings, whereas G bands were associated with the sp^2^-hybridized carbon atoms.^54^ Additionally, under the fitted curves the *T* (1424 cm^−1^) and D′′ (∼1490 cm^−1^) bands indicated the likely presence of disordered sp^2^–sp^3^ bonds and amorphous carbons in the SiOC fibers, respectively. However, for TPTS-derived SiOC sample, no pronounced D and G peaks were observed as a strong fluorescence background was produced under the visible laser source (HeNe 632.8 nm). The presence of carbon domains in the TPTS-SiOC fibers were later confirmed using XPS analysis.

**Fig. 5:**
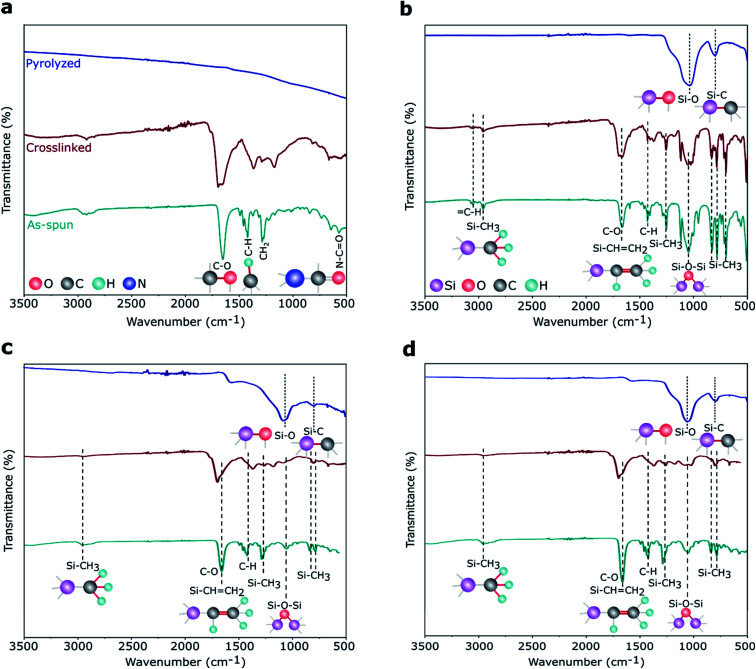
FTIR spectra of electrospun (a) PVP, (b) DDTS-, (c) DTDS-, and (d) TPTS-derived samples show the evolution of chemical bonds from the as-spun to pyrolyzed stages.

FTIR spectra, plotted in Fig. 5, shows the characteristic absorption bands of the PVP and polymer-to-ceramic conversion of preceramic Si precursors from the as-spun to pyrolyzed stages. For the PVP, as shown in Fig. 5a, as-spun and crosslinked samples exhibited the main peaks at 1660, 1427, 1291, and 570 cm^−1^, that were assigned to the stretching of C–O, C–H, C–H_2_, and N–C=O bonds, respectively.^55^ The presence of these peaks in both as-spun and crosslinked PVP fibers suggested no noticeable crosslinking happened during heat treatment at ∼300 °C, while after pyrolysis at ∼800 °C these peaks disappeared. DDTS fibers electrospun with PVP (as spinning agent) showed (Fig. 5b) Si–CH_3_ (∼1260 cm^−1^), and Si–O–Si (∼1060 cm^−1^) stretching bonds in the as-spun fibers indicating the obvious presence of Si precursors in these samples.^47,56^ Si–CH=CH_2_ (∼1660 cm^−1^) are superimposed with the C–O vibration. Reduction of Si–CH_3_ and Si–CH=CH_2_ peak intensities in the crosslinked samples suggested crosslinking reactions occurred at approx. 300 °C. After pyrolysis, the two obvious peaks at ∼1060 and ∼800 cm^−1^ confirmed the presence of Si–O and Si–C bonds, respectively, in the pyrolyzed SiOC fibers.^57^ DTDS- and TPTS-derived SiOC fibers also showed similar characteristic peaks from as-spun to pyrolyzed stages, which are presented in Fig. 5c and d. However, Si–O bonds were more intense than Si–C bonds, especially in DTDS-derived SiOC fibers (Fig. 5c), indicating the presence of more Si–O bonds as compared to Si–C bonds in the ceramic samples. Note the peaks around 3000–3100 cm^−1^ for DDTS, which can be attributed to =C–H stretching of the aromatic phenyl group.^58^

**Fig. 6:**
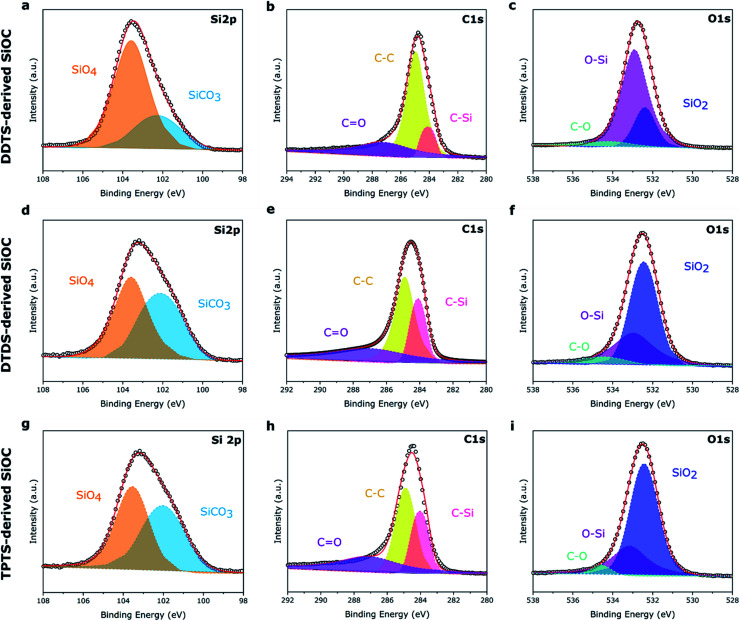
High-res XPS spectra of the three preceramic polymer-derived SiOC fiber samples. (a–c) DDTS-derived SiOC; (d–f) DTDS-derived SiOC; (g–i) TPTS-derived SiOC.

XPS survey spectra of the electrospun SiOC fibers, presented in Fig. S2,‡ showed similar Si 2p, C 1s, and O 1s peaks, irrespective of the source of the preceramic polymer. The surface composition of the elements was determined by integrating the area under the respective peaks and presented in Table 1. As expected, pyrolyzed PVP fibers mostly showed the presence of carbon (93.06 at%) after heat treatment. DDTS- and TPTS-derived ceramic fibers shows increased level of oxygen in the pyrolyzed samples indicating the inclusion of oxygen during crosslinking of the fibers. All specimens showed a significant amount of carbon, mainly free carbon, also proposed by Raman analysis. However, pyrolyzed samples presented a little amount of nitrogen, which was suspected to have come from PVP. High-resolution XPS spectra, plotted in Fig. 6, shows the bonding of the pyrolyzed SiOC samples derived from various siloxane precursors. Curve fittings were done to the Si 2p, C 1s, and O 1s peaks. The spectra under Si 2p band indicated the presence of SiCO_3_ (102.1 eV) and SiO_4_ (103.5 eV) peaks.^31^ Low intensity of SiCO_3_ compared to SiO_4_ for pyrolyzed-DDTS ceramic fibers were due to the low carbon content in the elemental composition. In addition, C–Si (284.0 eV), C–C (284.9 eV), and C=O (287.1 eV) were observed in C 1s band.^47^ The higher amount of carbon in the pyrolyzed-DTDS and pyrolyzed-TPTS fibers lead to higher intensity of C–C and C–Si peaks. The intense C–C peaks also pointed to increased possibility of free carbon phase in the ceramic fibers. O 1s band were fitted with 3 peaks at 532.4, 532.9, and 534.4 eV corresponding to SiO_2_, O–Si, and C–O, respectively.

**Table 1:** Elemental composition by XPS

Pyrolyzed samples	Elements (at%)			
	C	O	Si	N
Pyrolyzed PVP	93.06	4.84	—	2.10
DDTS-derived SiOC	32.35	37.58	29.70	0.37
DTDS-derived SiOC	73.80	9.42	16.09	0.68
TPTS-derived SiOC	58.00	19.28	21.74	0.98

**Fig. 7:**
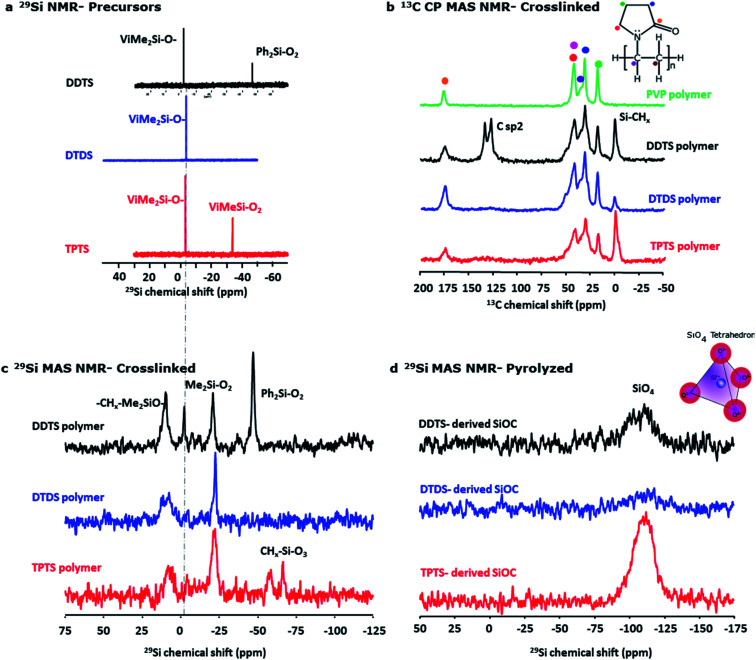
NMR spectra of precursors, crosslinked and pyrolyzed samples. (a) ^29^Si liquid state NMR of the electrospinning solution, (b) comparison of solid state ^29^Si MAS NMR spectra of crosslinked polymers of various preceramic Si precursors, (c) ^13^C CP MAS NMR spectra of crosslinked (at ∼300 °C) polymers in comparison with PVP, (d) comparison of ^29^Si MAS NMR spectra of pyrolyzed (at ∼800 °C) ceramic powders.

The initial composition of the electrospinning solution was investigated by liquid state NMR. ^29^Si spectra (Fig. 7a) show for all systems a signal around −3 ppm corresponding to ViMe_2_SiO environments. In addition, signals are observed at −46.8 ppm and −34.0 ppm in DDTS and TPTS based solutions that can respectively be assigned to Ph_2_SiO_2_ and ViMeSiO_2_ groups. This assignment was confirmed by 2D experiments. For example, ^1^H–^29^Si HMBC map of DDTS solution (Fig. S3a‡) shows that the ^29^Si signal at −47 ppm shows cross-peaks with ^1^H phenyl signals between 7.5 and 8 ppm while the ^29^Si signal at −34 ppm shows cross-peaks with ^1^H vinyl signals between 6 and 5.5 ppm. The evolution of the various preceramic siloxane precursor structures during heat treatment and the resulting SiOC samples were also investigated using solid-state NMR. Spectra were recorded on bulk powders instead of fibers to improve the signal/noise ratio.

Fig. 7b shows ^29^Si MAS NMR spectra for crosslinked (at ∼300 °C) polymers and compares the key features of the DDTS, DTDS, and TPTS polymers. Appearance of –CH_*x*_–Me_2_SiO (∼10 ppm) in the polymers indicated effective crosslinking of the vinyl groups occurred in presence of DCP.^59^ On the other hand, a signal appeared at −21 ppm which was characteristic of Me_2_SiO_2_ units in polydimethylsiloxane (PDMS) chains.^60,61^ This would suggest that part of the Si–CH=CH_2_ groups were replaced by Si–O–Si bonds during heat-treatment as confirmed by the total disappearance of ViMeSiO_2_ units at −3 ppm in DTDS and TPTS cross-linked systems. Moreover, in the case of TPTS, ViMeSiO_2_ units observed at −34 ppm in the initial solution disappeared in the cross-linked system while new signals were present around −70 ppm, a chemical shift range characteristic of MeSiO_3_ environments. Here again, this suggested the replacement of Si–CH=CH_2_ groups by Si–O–Si bonds. In the case of DDTS, Me_2_SiO_2_ units were observed but ViMeSiO_2_ were also present in small quantity in accordance with *iR* experiments showing remaining vinyl groups after crosslinking (Fig. 5b). The signal corresponding to Ph_2_SiO_2_ units (−48 ppm) was still intense after crosslinking suggesting that Si–Ph bonds were preserved. The ^29^Si MAS spectra of the samples pyrolyzed at 800 °C showed SiO_4_ bonds (−110.0 ppm) (Fig. 7d), while no Si–C bonds were found.^62^ This might be due to the fact mostly free C phase had formed during pyrolysis, and very low Si–C bonds were present in the ceramic samples.^47^ Nevertheless, high-res XPS of the SiOC fibers indicated the presence of Si–C and C–C bonds in the ceramic fibers.

^13^C cross polarization (CP) MAS NMR spectra in Fig. 7c show strong peaks of Si–CH_*x*_ indicating the crosslinking of the Si precursor polymers. In the DTDS polymer, sp^2^ carbons were observed around 130 ppm that corresponded mainly to the phenyl groups. Indeed, from ^1^H–^13^C HSQC map of DDTS solution (Fig. S3b‡) cross-peaks with ^1^H phenyl signals (between 7.5 and 8 ppm) were observed for carbon signals at 127.6, 129.7 and 134.4 ppm, while the cross-peaks with ^1^H vinyl signals (between 6 and 5.5 ppm) were observed with carbon signals at 132.1 and 139.0 ppm. The absence of a clear ^13^C peak at 139 ppm in the DTDS cross-linked sample suggested that the proportion of vinyl groups was small compared to phenyl groups. No evidence of crosslinking behavior between PVP molecules and siloxane was observed in all the crosslinked samples when compared with PVP polymer. ^13^C CP MAS NMR spectrum of the TPTS-derived SiOC confirmed the presence of typical free carbon as shown in Fig. S4.‡^13^C spectra of the remaining SiOC samples were not acquired as it was obvious from the other characterization techniques that all the systems were full of graphite.

### 3.3 Electrochemical performance

**Fig. 8:**
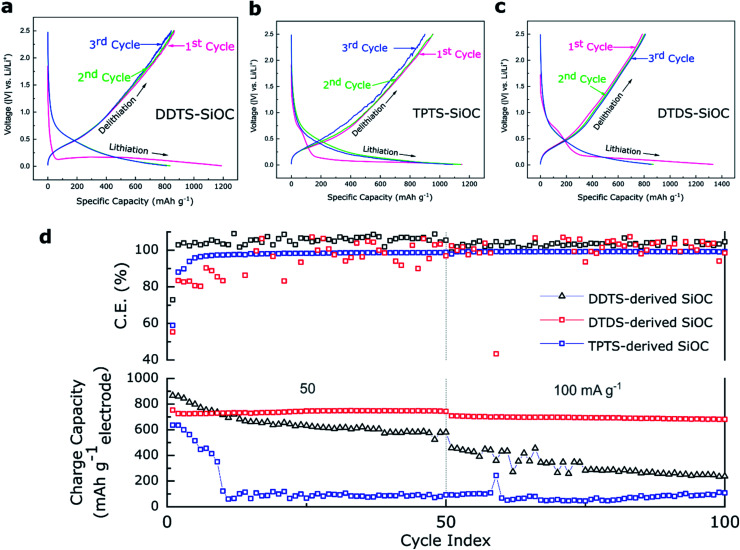
Charge-storage performances of the various precursor-derived SiOC freestanding fibermats in the Li half-cells. (a–c) Voltage profile of the SiOC electrodes show the charge–discharge profiles for the first three cycles, (d) cycling stability data of the samples for 100 cycles. DTDS-SiOC shows stable performance delivering 680 mA h g^−1^ at 100 mA g^−1^, whereas DDTS-SiOC and TPTS-SiOC deliver 240 and 110 mA h g^−1^, respectively after 100 cycles.

The electrochemical energy storage capability of the precursor derived SiOC fibers were analyzed in Li half-cells. The as-prepared ceramic fibermats were used as electrodes. Fig. 8a–c shows the potential *vs.* capacity plots for various SiOC fiber electrodes. A closer look at the charge–discharge profiles of the samples at a current density of 50 mA g^−1^ showed that first cycle had experienced irreversible capacity decay. Among the SiOC electrodes, the DDTS-derived SiOC delivered initial discharge capacity of 1188 and charge capacities of 866 mA h g^−1^, corresponding to a high coulombic efficiency of over 72%. DTDS-derived SiOC electrode delivered 1332 and 800 mA h g^−1^, respectively for discharge and charge capacities in the first cycle with 59% efficiency. TPTS-derived SiOC provided 1150 and 636 mA h g^−1^ for discharge and charge capacities, respectively, with 52% coulombic efficiency. DDTS- and DTDS-SiOC electrodes showed stable response with coulombic efficiency of ∼100% in the second and third cycles, whereas TPTS-SiOC displayed poor reversibility with only ∼83% efficiency. Differential capacity plots, presented in Fig. S5,‡ showed two distinct regions for the SiOC electrodes: a sharp peak around 0.1 V, which corresponded to the Li insertion into the carbons; and a broad region in between 0.2–0.6 V, that was related to Li and Si–O–C phase.^27^

Accordingly, when tested for cycling stability TPTS-SiOC electrode showed poor performance and the charge capacity dropped sharply to 100 mA h g^−1^ after 50 cycles at 50 mA g^−1^. For DDTS- and DTDS-SiOC electrodes, the capacities after 50 cycles were 580 and 800 mA h g^−1^, showing 100% capacity retention for the DTDS-derived SiOC fiber electrode. The distinct fiber morphology and the “free C“ content in the DTDS-SiOC particles provided additional Li-ion storing sights and improved the electrochemical properties of SiOC fibers.^63^ The rate capability performance of the various SiOC electrodes is presented in Fig. S5.‡ DTDS-SiOC delivered a high capacity of 450 mA h g^−1^ at a high current density of 800 mA g^−1^. However, thin diameter and hollow structure of the TPTS-SiOC fibers might have been destroyed during Li-ion insertion/extraction process, which resulted in poor cycleability and rate capability.^64^ The SEM and TEM images of the SiOC electrodes after cycling in LIBs are shown in Fig. S7,‡ where the broken fibers of the TPTS-SiOC can be seen post-cycling. Whereas poor rate capability performance of the DDTS-SiOC can be attributed to the low C content as well as dense structure of the fibers which limits the ion diffusion. To show the consistent performance of the cells, three Li half-cells of each type were assembled and tested and the performance of all the cells are presented in Fig. S5.‡ Fig. S5(a–c)‡ shows the differential capacity curves of the SiOC electrodes for the initial 3 cycles. The sharp cathodic peak at 0.005 V and the broad anodic peak can be attributed to the lithiation and delithiation of Li^+^ ions in the amorphous SiOC structure.^54,65^ The cathodic peaks in the range of 0.05–0.16 V can be attributed to the irreversible reaction in hard carbon present in the SiOC structure.^25,65^ However, cathodic peaks due to formation of SEI (typically at ∼0.5 V) are less pronounced in the d*Q*/d*V* plots. A comparison of the electrochemical performance of the polymer-derived SiOC anodes with reported SiOC anodes for LIBs is presented in Table S1.‡

**Fig. 9:**
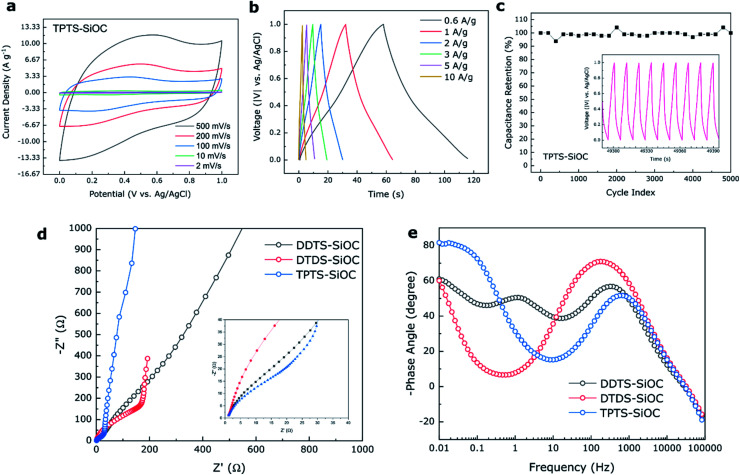
Electrochemical performance of TPTS-derived SiOC supercapacitor electrode in 1 M H_2_SO_4_ aqueous electrolyte. (a) CV profile of the electrode at various scan rate; (b) GCD curve of the electrode at various current density; (c) cycling performance of the TPTS-derived SiOC electrode over 5000 cycles shows 100% capacitance retention; (d) comparison of impedance spectra among the various SiOC electrodes; (e) comparison of Bode plots indicating variation of phase angles among the electrodes.

The electrochemical storage capabilities of SiOC fibers in supercapacitors were also investigated using three electrode system and 1 M H_2_SO_4_ as electrolyte. CV plots obtained for TPTS-derived SiOC electrode at various scan rates of 2 to 500 mV s^−1^ from 0 to 1 V are shown in Fig. 9a. The almost rectangular shape of the voltammogram profiles indicated mostly the electrochemical double layer capacitive behavior (Type A) of the SiOC electrode during charge storage.^66^ However, at a low scan rate of 2 mV s^−1^, a broad redox peak was observed for TPTS-SiOC electrode in between 0.2 and 0.4 V (Fig. S6d‡). The charge storage mechanism at lower scan rate can be contributed to the combination of double-layer capacitance of C in SiOC and pseudocapacitance from Si–O and Si–Si components. The quasi-rectangular shape of the CV plot retained even at higher scan rate of 500 mV s^−1^, indicating higher ionic diffusivity and charge transfer of the TPTS-SiOC electrode. As a result, the electrode delivered high specific capacitances of 78, 69, 55, 47, and 38 F g^−1^ at the scan rates of 2, 10, 100, 200, and 500 mV s^−1^. The areal capacitance of the TPTS-SiOC electrode was also calculated and a high capacitance of 474 mF cm^−2^ was achieved at 2 mV s^−1^. The cyclic voltammograms of DTDS- and DDTS-derived SiOC supercapacitor electrodes are presented in Fig. S6.‡ DTDS-SiOC delivered 107 F g^−1^ at a scan rate of 2 mV s^−1^ and decreased to 31 F g^−1^ at 100 mV s^−1^, where DDTS-SiOC delivered 25 and 3 F g^−1^ at 2 and 100 mV s^−1^, respectively. The highest performance of TPTS-SiOC among the three SiOC electrodes was correlated to the lowest diameter and hollow core of the fibers, contributing to a higher electrochemically active area for double-layer capacitor. The BET analysis and avg. pore diameter of the SiOC samples are presented in ESI Section 7.‡ As-anticipated the TPTS-SiOC had a high specific surface area of 235 m^2^ g^−1^. In addition, the presence of “free C” contributed to the enhanced electronic conductivity. Fig. 9b demonstrates the charge/discharge profiles of the best performing TPTS-SiOC electrodes at various current densities from 0 to 1 V. The quasi-triangular shape of the GCD curves without any obvious plateau suggested the dominating double-layer capacitive behavior of the electrode, which was in accord with earlier results.^67^ The triangular shape of the GCD is a typical behavior of highly reversible supercapacitor with constant charge/discharge.^68^ As a result, TPTS-SiOC delivered 30 F g^−1^ even at a high current density of 10 A g^−1^. When tested for cycling stability, the electrode demonstrated ∼100% capacitance retention over 5000 cycles at 3 A g^−1^. DDTS- and DTDS-derived SiOC electrodes also showed stable cycling ability over 5000 cycles as shown in Fig. S6.‡

EIS was done for the various SiOC electrodes to further understand the reaction kinetics of the supercapacitor samples. In Fig. 9d, the ohmic resistance, *R*_s_ between the aqueous electrolyte and the electrode was measured in the high frequency region where the curves intercept the *Z*′ axis. The semicircle in medium frequency region was attributed to the charge transfer resistance (*R*_CT_) of the electrolyte–electrode interface, and the inclined lines in the low frequency region corresponded to the ion diffusion into the SiOC electrode materials.^54,69^ An equivalent circuit was obtained for the electrodes, as shown in Fig. S6,‡ from which the calculated *R*_CT_ for TPTS-SiOC electrode (6 Ω) was much lower than the DDTS-SiOC (15 Ω) and DTDS-SiOC (50 Ω) electrodes. In the low frequency region, the near vertical line to the real axis of the TPTS-SiOC electrode corresponded to the ideal capacitive behavior.^70^ Furthermore, the Bode plot (Fig. 9e) showed that TPTS-SiOC had the nearest phase angle (80.5°) to 90°, indicating the best capacitive behavior among the SiOC electrodes.^71^ At phase angle of 45°, the relaxation time constant, *τ*_o_ (*τ*_o_ = 1/*f*_o_) were measured to be 2.61, 6.81, and 56.18 ms for the TPTS-SiOC, DDTS-SiOC, and DTDS-SiOC electrodes, respectively. The significantly lower time constant of the TPTS-SiOC electrode confirmed the fast ion diffusion and transport characteristic as a supercapacitor electrode.

## 4. Conclusion

In summary, we have presented a method to fabricate scalable, self-supporting electrodes from precursor-based SiOC fibers utilizing three unique siloxane precursors. The electrospinning of the short-chain siloxane oligomers was not achievable without the addition of a spinning agent (such as PVP). In addition, the precursor-to-ceramic yield of the fibers was also observed to be lower than some of the previously reported polymer-derived ceramic materials. This phenomenon suggested limited thermal crosslinking of the fibers in the presence of 1 wt% DCP. The electron microscopy of the SiOC fibers featured rigid surface structures and small diameters (0.2–3 μm). Raman, XPS, FTIR, and NMR characterization techniques outlined the polymer to ceramic conversion stages. ^29^Si MAS NMR spectra of the SiOC fibers showed only SiO_4_ bonds, indicating mostly free C phase had formed during pyrolysis with a low amount of Si–C bonds in the ceramic samples. The amorphous SiOC structures, comprised of free carbon phase and Si–O–C mixed bonds of DTDS-derived SiOC contributed to high reversibility of Li storage. The free carbon phase served as electron conductor as well as the major electrochemically active sites for Li ions, while amorphous Si–O–C network contributed to minor Li storage. In terms of electrochemical properties, the SiOC electrodes displayed excellent capacity with a high coulombic efficiency in LIBs. As supercapacitor electrodes, superior cycleability of 100% capacitance retention over 5000 cycles was achieved. The as-prepared SiOC fiber mats can provide highly efficient, high-energy, and high-power electrodes and reduce the total weight of the electrochemical energy storage devices.

## Author contributions

S. M. carried out the preparation of all samples, perform characterization, data analysis and drafted the manuscript; F. R. and C. G. contributed to perform NMR characterizations, data analysis and drafting of the manuscript; G. S. conceived the idea, designed the project and helped with drafting the manuscript.

## Conflicts of interest

There are no conflicts of interest.

## Supplementary Material

RA-011-D1RA05968H-s001
