# Supplementary material for: Self-supporting carbon-rich SiOC ceramic electrodes for lithium-ion batteries and aqueous supercapacitors
Source: RSC Adv. 2021 Nov 3;11(56):35440–54. doi: 10.1039/d1ra05968h (PMC9042965; doi:10.1039/d1ra05968h)
Supplement: RA-011-D1RA05968H-s001 [file RA-011-D1RA05968H-s001.pdf]

Electronic Supplementary Material (ESI) for RSC Advances.
This journal is © The Royal Society of Chemistry 2021

Self-supporting Carbon-rich SiOC Ceramic Electrodes for Lithium-ion Batteries and Aqueous Supercapacitors

Shakir Bin Mujib, François Ribot, Christel Gervais, Gurpreet Singh

Supplementary Information

1. High-resolution TEM and EDX

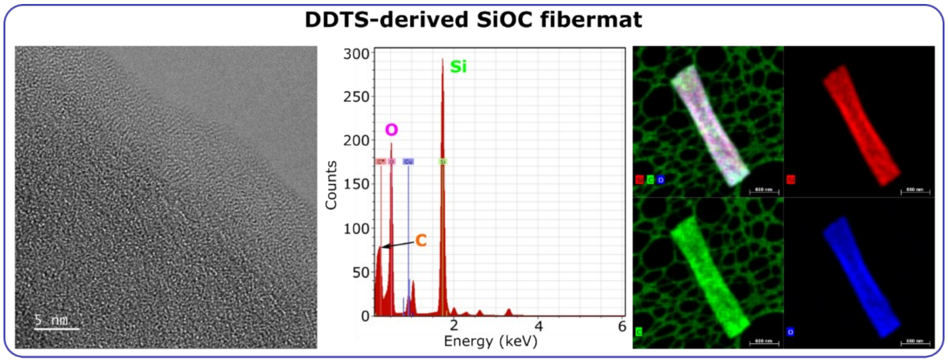

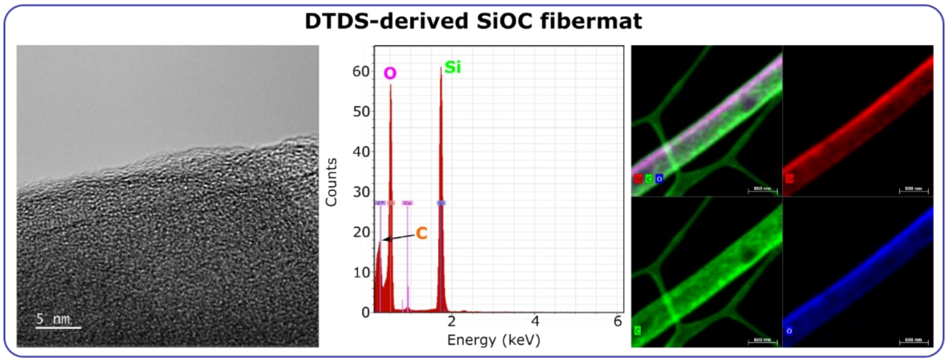

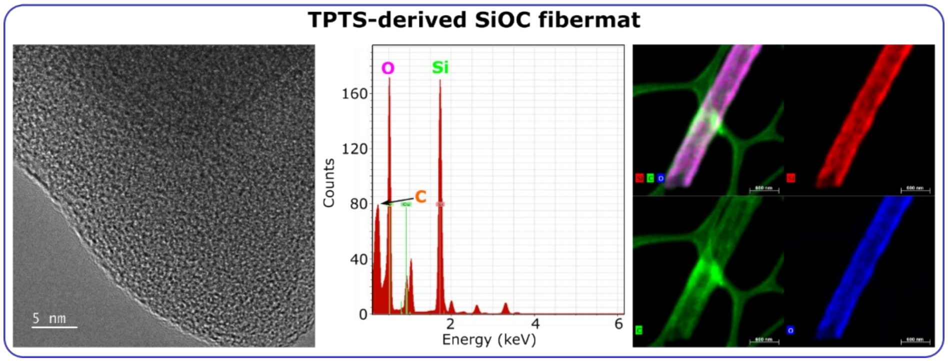

Figure S1: High-res TEM and corresponding EDX mappings of the SiOC fibermats. High-res TEM reveals the amorphous structure of the PDC fibermats. EDX elemental analysis show the presence and homogenous distribution of Si, C, and O in the three polymer-derived SiOC fibermats.

2. XPS

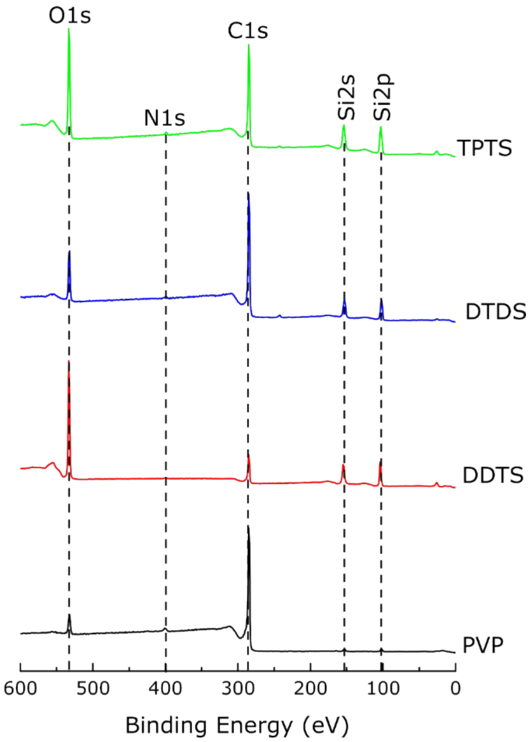

Figure S2: XPS spectra of the various samples pyrolyzed at 800 °C.

3. NMR

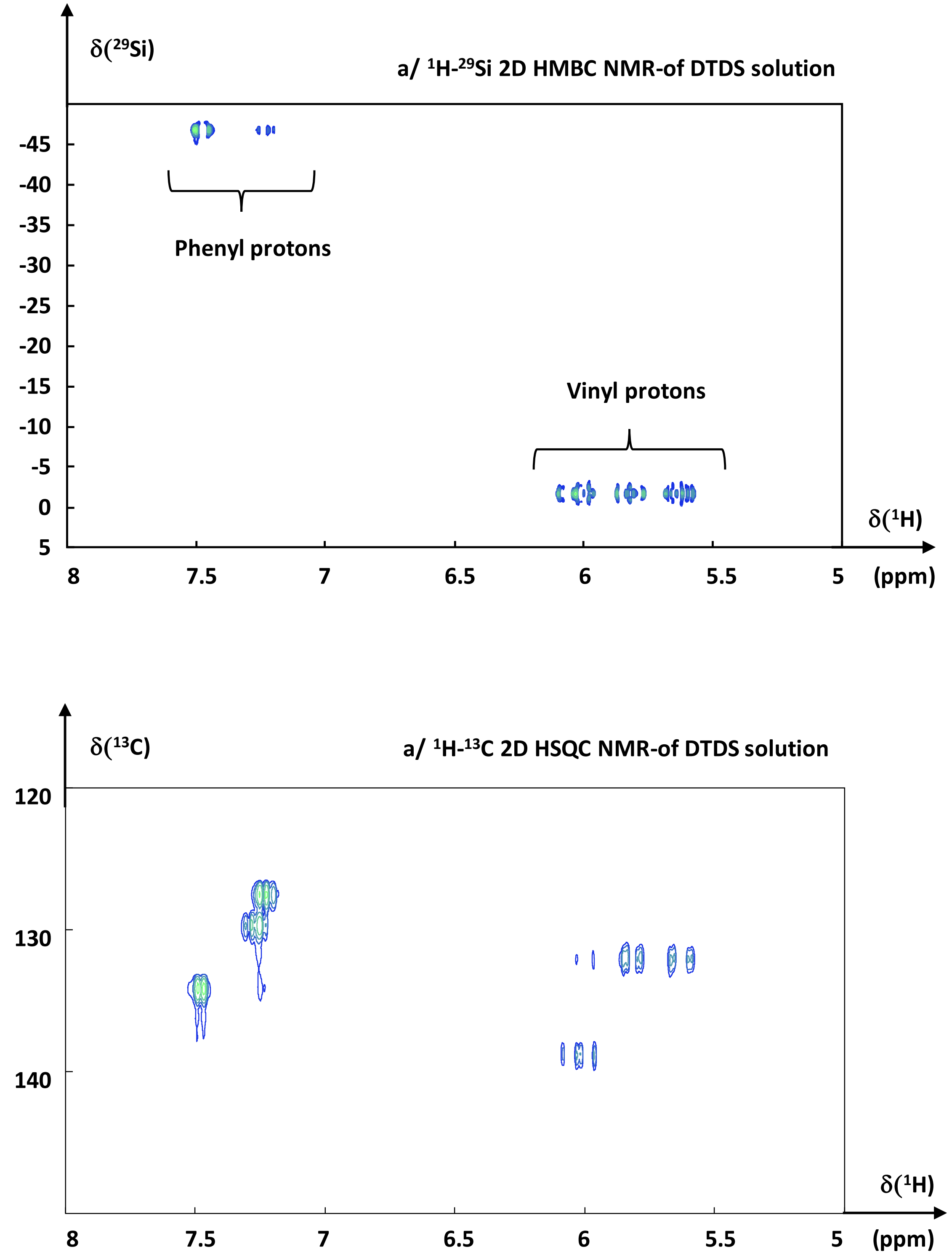

Figure S3: (a) ^{1}H-^{29}SiC and (b) ^{1}H-^{13}C 2D NMR spectra of the DTDS electrospinning solution.

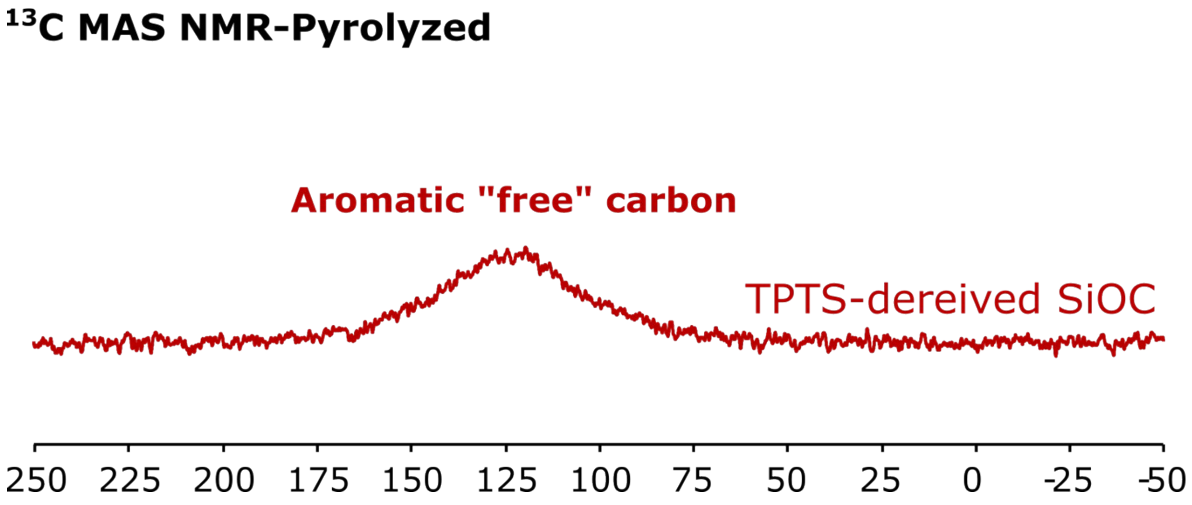

Figure S4: ^{13}C CP MAS NMR spectrum of pyrolyzed (at ~800 °C) TPTS-derived SiOC ceramic powders indicates the presence of typical "free carbons".

4. LIBs

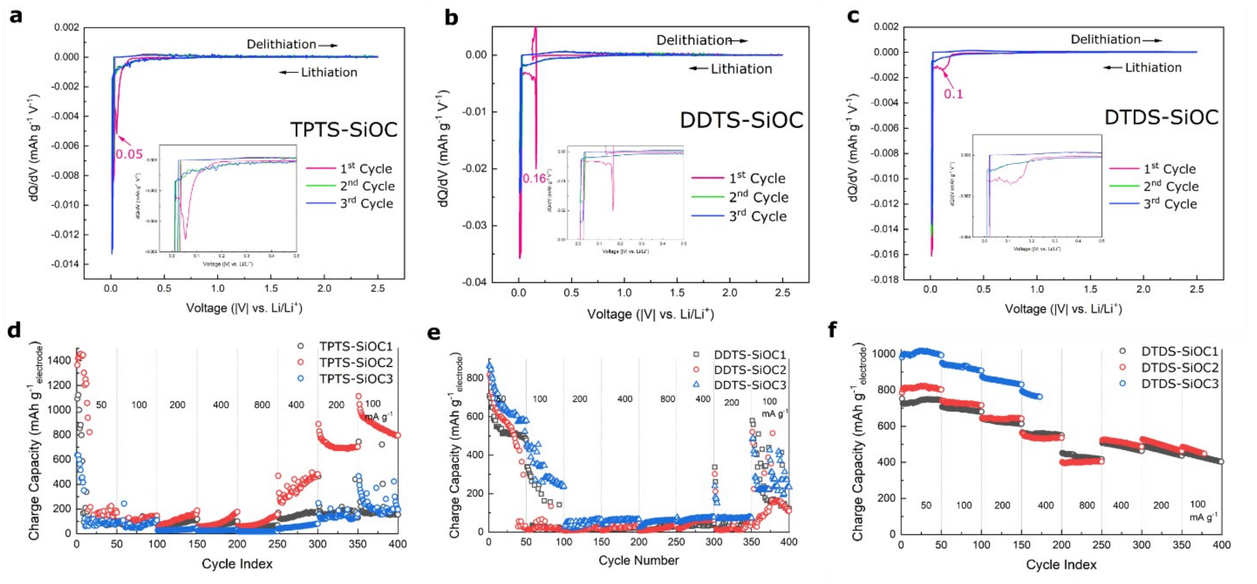

Figure S5: (a-c) Differential capacity curves of various SiOC electrodes show characteristic regions of reversible reactions between Li and SiOC. (d-f) Rate capability performance of three SiOC electrodes of each type shows consistent performance.

Table S1: Comparison of the electrochemical performance of the polymer-derived SiOC anode with reported SiOC anodes for LIBs

Samples	Current density ($mA\ g^{-1}$)	Cycles	Capacity ($mAh\ g^{-1}$)	Ref.
SiOC/C	50	60	669	[1]
SiOC/graphene	50	90	582	[2]
SiOC	74	30	460	[3]
SiOC/graphene sponge	100	100	701	[4]
SiOC	36	30	~150	[5]
Si/SiOC	100	100	800	[6]
SiOC/C NFs	100	100	707	[7]
SiOC/N-doped C	200	200	595	[8]
Si/SiOC/graphie	0.3C	100	637.3	[9]
3D-GNS/$SiOC_f$	100	100	775	[10]
SiOC fibermat	50	50	800	**This work**
SiOC fibermat	800	250	450	**This work**

5. Supercapacitors

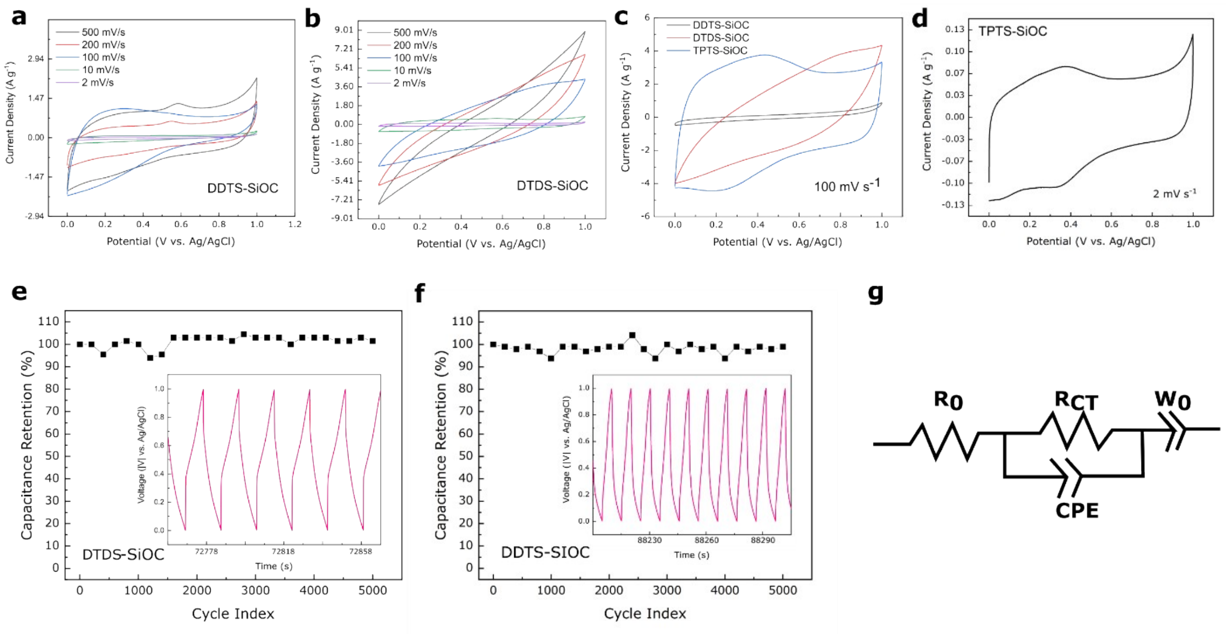

Figure S6: (a-b) CV profiles of the DDTS- and DTDS-derived SiOC supercapacitor electrodes at various scan rate in 1M H_2SO_4 aqueous electrolyte, (c) comparison of cyclic voltammograms of various electrodes at 100 mV s^{-1}, (d) TPTS-derived SiOC supercapacitor electrodes at a scan rate of 2 mV s^{-1}, (e-f) capacitance retention trend of DTDS- and DDTS-derived SiOC electrodes show ~100% retention over 5000 cycles, (g) equivalent circuit derived for the supercapacitor electrodes.

6. Post-cycle analysis (SEM and TEM)

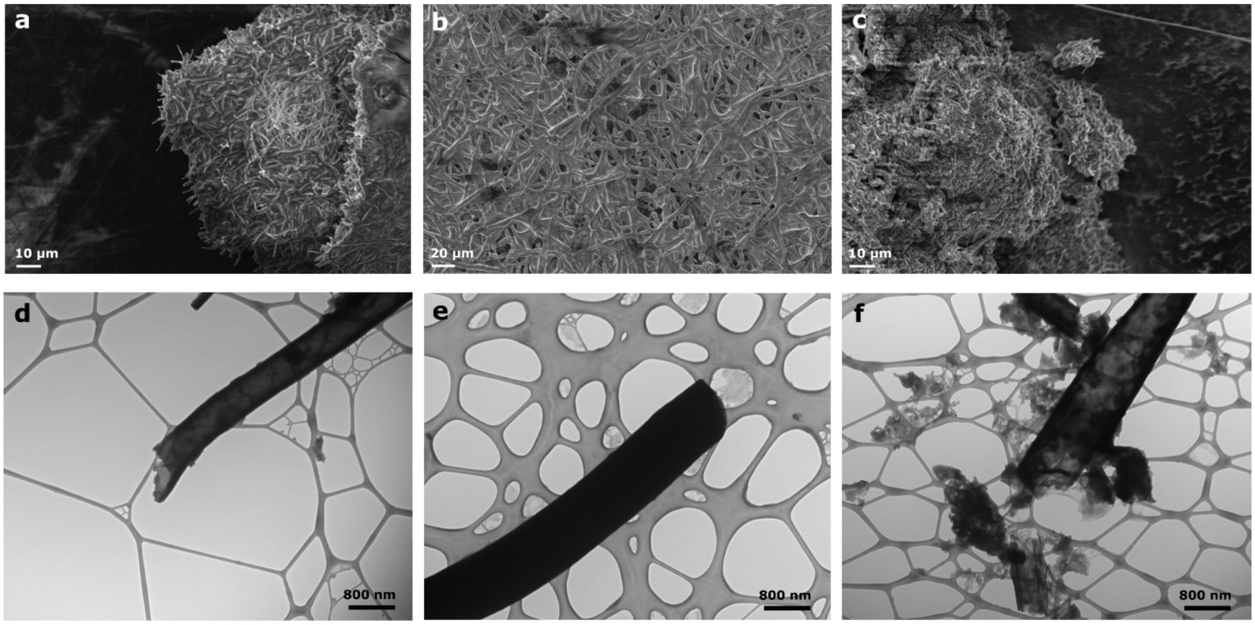

DTDS-derived SiOC **DDTS-derived SiOC** **TPTS-derived SiOC**

Figure S7: Microscopy analysis of various SiOC electrodes after 400 cycles in LIBs. (a-c) SEM images and (d-f) TEM images. TEM image of TPTS-SiOC shows the broken fiber structure presumably due to the lithiation/delithiation.

7. BET Surface Area Analysis

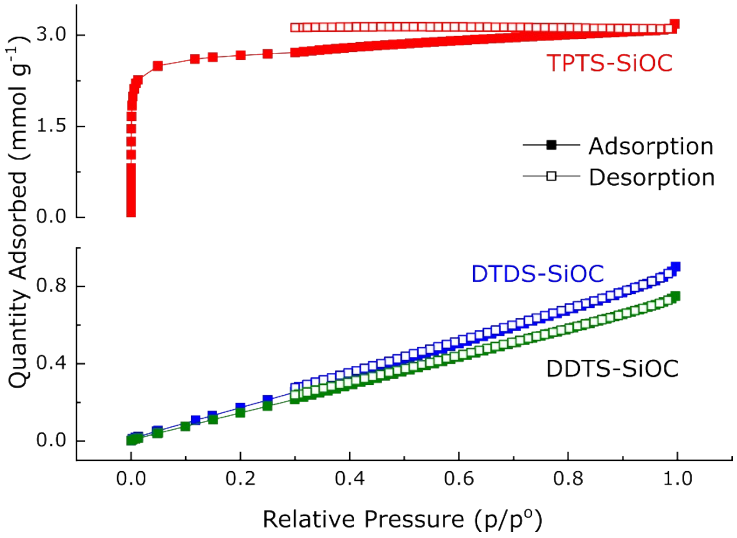

Figure S8: Nitrogen adsorption-desorption isotherms for the BET surface area analysis of various SiOC fibers.

Table S2: BET analysis results

Samples	BET surface area (m^2 g^{-1})	Adsorption avg. pore diameter (Å)
TPTS-derived SiOC	235	18.78
DTDS-derived SiOC	29	42.46
DDTS-derived SiOC	26	39.33

Reference

1. Li, Y.; Hu, Y.; Lu, Y.; Zhang, S.; Xu, G.; Fu, K.; Li, S.; Chen, C.; Zhou, L.; Xia, X.; Zhang, X., One-dimensional SiOC/C composite nanofibers as binder-free anodes for lithium-ion batteries. *Journal of Power Sources* **2014,** *254*, 33-38.
2. Ren, Y.; Yang, B.; Huang, X.; Chu, F.; Qiu, J.; Ding, J., Intercalated SiOC/graphene composites as anode material for li-ion batteries. *Solid State Ionics* **2015,** *278*, 198-202.
3. Kaspar, J.; Graczyk-Zajac, M.; Choudhury, S.; Riedel, R., Impact of the electrical conductivity on the lithium capacity of polymer-derived silicon oxycarbide (SiOC) ceramics. *Electrochimica Acta* **2016,** *216*, 196-202.
4. Sang, Z.; Zhao, Z.; Su, D.; Miao, P.; Zhang, F.; Ji, H.; Yan, X., SiOC nanolayer wrapped 3D interconnected graphene sponge as a high-performance anode for lithium ion batteries. *Journal of Materials Chemistry A* **2018,** *6* (19), 9064-9073.
5. Dibandjo, P.; Graczyk-Zajac, M.; Riedel, R.; Pradeep, V. S.; Soraru, G. D., Lithium insertion into dense and porous carbon-rich polymer-derived SiOC ceramics. *Journal of the European Ceramic Society* **2012,** *32* (10), 2495-2503.
6. Wu, Z.; Lv, W.; Cheng, X.; Gao, J.; Qian, Z.; Tian, D.; Li, J.; He, W.; Yang, C., A Nanostructured Si/SiOC Composite Anode with Volume-Change-Buffering Microstructure for Lithium-Ion Batteries. *Chemistry – A European Journal* **2019,** *25* (10), 2604-2609.
7. Huang, X.; Christopher, B.; Chai, S.; Xie, X.; Luo, S.; Liang, S.; Pan, A., Cowpea-like N-Doped Silicon Oxycarbide/Carbon Nanofibers as Anodes for High-Performance Lithium-Ion Batteries. *ACS Applied Energy Materials* **2021,** *4* (2), 1677-1686.
8. Ma, M.; Wang, H.; Li, X.; Peng, K.; Xiong, L.; Du, X., Free-standing SiOC/nitrogen-doped carbon fibers with highly capacitive Li storage. *Journal of the European Ceramic Society* **2020,** *40* (15), 5238-5246.
9. WANG Jian-Tao, W. Y., HUANG Bin, YANG Juan-Yu, TAN Ao, LU Shi-Gang, Silicon Supported on Stable Si-O-C Skeleton in High-Performance Lithium-Ion Battery Anode Materials. *Acta Phys. -Chim. Sin.* **2014,** *30* (2), 305-310.
10. Sang, Z.; Yan, X.; Wen, L.; Su, D.; Zhao, Z.; Liu, Y.; Ji, H.; Liang, J.; Dou, S. X., A graphene-modified flexible SiOC ceramic cloth for high-performance lithium storage. *Energy Storage Materials* **2020,** *25*, 876-884.
